# Selection on MHC class II supertypes in the New Zealand endemic Hochstetter’s frog

**DOI:** 10.1186/s12862-015-0342-0

**Published:** 2015-04-13

**Authors:** Mette Lillie, Catherine E Grueber, Jolene T Sutton, Robyn Howitt, Phillip J Bishop, Dianne Gleeson, Katherine Belov

**Affiliations:** Faculty of Veterinary Science, University of Sydney, Sydney, Australia; San Diego Zoo Global, San Diego, USA; Department of Biology, University of Hawai’i at Mānoa, Honolulu, USA; Landcare Research, Auckland, New Zealand; Department of Biology, University of Otago, Dunedin, New Zealand; Institute for Applied Ecology, University of Canberra, Bruce, Australia

**Keywords:** Conservation genetics, *Leiopelma hochstetteri*, Fragmentation, Balancing selection, Genetic drift

## Abstract

**Background:**

The New Zealand native frogs, family Leiopelmatidae, are among the most archaic in the world. *Leiopelma hochstetteri* (Hochstetter’s frog) is a small, semi-aquatic frog with numerous, fragmented populations scattered across New Zealand’s North Island. We characterized a major histocompatibility complex (MHC) class II B gene (DAB) in *L. hochstetteri* from a spleen transcriptome, and then compared its diversity to neutral microsatellite markers to assess the adaptive genetic diversity of five populations (“evolutionarily significant units”, ESUs).

**Results:**

*L. hochstetteri* possessed very high MHC diversity, with 74 DAB alleles characterized. Extremely high differentiation was observed at the DAB locus, with only two alleles shared between populations, a pattern that was not reflected in the microsatellites. Clustering analysis on putative peptide binding residues of the DAB alleles indicated four functional supertypes, all of which were represented in 4 of 5 populations, albeit at different frequencies. Otawa was an exception to these observations, with only two DAB alleles present.

**Conclusions:**

This study of MHC diversity highlights extreme population differentiation at this functional locus. Supertype differentiation was high among populations, suggesting spatial and/or temporal variation in selection pressures. Low DAB diversity in Otawa may limit this population’s adaptive potential to future pathogenic challenges.

**Electronic supplementary material:**

The online version of this article (doi:10.1186/s12862-015-0342-0) contains supplementary material, which is available to authorized users.

## Background

**Figure 1:**
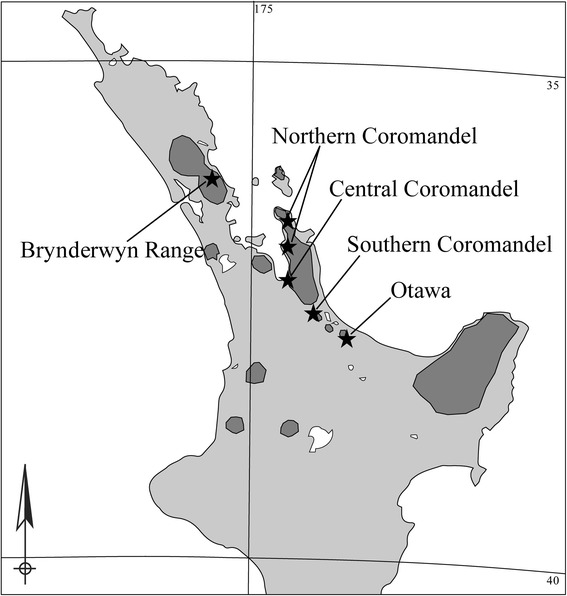
Map of New Zealand North Island showing *Leiopelma hochstetteri* distribution (dark gray shading) and sampling locations (white star represents the sample location of the individual euthanized for the spleen transcriptome; black stars indicate sampling locations included in population study).

The frog family Leiopelmatidae contains four species of the genus *Leiopelma* [1]. These frogs are among the most archaic in the world [2] and only found in New Zealand. *Leiopelma hochstetteri* is a small, semi-aquatic species [3]. It is the most widespread and common species within this genus, but populations are fragmented and scattered over an extensive area of the North Island (Figure 1) [4,5]. Sub-fossil evidence indicates that *L. hochstetteri* was historically more widely distributed throughout the North Island and in the northern half of the South Island [6]. The introduction of mammalian predators and habitat modifications following human settlement of New Zealand in the 17th century have been major contributors to the modern-day fragmentation and population declines [6].

Populations show significant genetic and cytogenetic distinctiveness [7-9], with 13 evolutionarily significant units (ESUs) defined using mitochondrial and nuclear genetic markers [10]. Molecular dating has estimated that this genetic differentiation originated from the early Pleistocene, a geographically turbulent period in New Zealand that would have impacted population connectively [10]. The overall population size of *L. hochstetteri* is estimated at greater than 100,000 mature individuals, but a population decline of at least 10% in total population or area of occupancy over the next three generations has been predicted [5]. As such*, L. hochstetteri* has been classified as “Vulnerable” on the IUCN Red List of Threatened Species [11] and as ‘At Risk: Declining’ under the New Zealand threat status criterion [5]. In its current fragmented state, *L. hochstetteri* faces many threats, most significantly the alteration of rocky stream ecosystems by land management processes such as logging, farming or by feral stock, as well as predation by introduced mammals [12-14].

High population structuring in *L. hochstetteri*, combined with these threatening processes, has significant conservation implications [5,10]. Previous genetic studies employed a variety of neutral genetic markers, but information on functional diversity is lacking. In the current study, we characterize diversity in an adaptive genetic region, the major histocompatibility complex (MHC). The MHC is a large gene family with a vital role in the vertebrate immune response [15]. The class II molecule is a heterodimer formed by an α chain and a β chain that is expressed on the surface of antigen-presenting cells [15]. MHC class II molecules present extracellular peptides to T-helper cells, with residues encoded by the α1 and β1 domains contributing to peptide recognition and binding [15]. These domains interact with extracellular peptides, such as those derived from bacterial, parasitic or fungal pathogens, and are usually highly polymorphic, driven by pathogen-mediated selection and mate-choice [16-20].

In natural populations, diversity of functional genomic regions, such as the MHC, is affected by both selective and neutral evolutionary forces. By contrast, non-functional genetic regions will reflect primarily only neutral forces, such as genetic drift and gene flow. Comparing functional and neutral diversity allow researchers to infer the relative influence of selection on this adaptive gene region [21-25]. For conservation aims, MHC has increasingly been used as an indicator of adaptive genetic variation, and has been recently employed to evaluate immunogenetic health [26], delineate conservation units [27], evaluate genetic restoration [28], and evaluate the genetic impacts of translocations [29]. In its application to infer immunogenetic health, populations with high MHC diversity may be better able to adapt to future pathogenic challenges as the chance of resistance alleles being present is greater [19], thus with decreased potential extinction risk relative to less-diverse populations. This conjecture has raised some debate [25], with multiple examples of long-term survival of populations with low MHC diversity [30-32]. Nevertheless, multiple empirical examples of associations between MHC variation and disease susceptibility [33-35] highlight the potential susceptibility of populations of low MHC diversity to disease epidemics.

In this study, we generated a *L. hochstetteri* spleen transcriptome to identify a MHC class II B gene (DAB). We then characterized diversity in the β1 domain (exon 2) and compared the results to diversity from nine microsatellite markers across five ESUs. Our results showed high MHC polymorphism with extremely high differentiation between studied ESUs, a pattern that was not reflected in the microsatellites.

## Methods

### Samples

One *L. hochstetteri* individual (collected from a mountain stream in the Pukeamaru region on the east coast of the North Island, New Zealand, 37°38“S, 178°15”E, Figure 1) was sacrificed for the spleen transcriptome preparation under Department of Conservation Authority OT-29713-FAU. Spleen tissue was collected and fixed in liquid nitrogen and subsequently stored at −80°C. The tissue was disrupted under liquid nitrogen in a mortar and pestle then RNA extracted using TRIzol reagent (Invitrogen) according to manufacturer’s instructions. RNA quality was assessed on a 2100 Bioanalyzer (Agilent Technologies Genomics) and extractions were stored at −80°C.

Toe clip samples for genetic analyses were collected under ethics approval granted by the Department of Conservation New Zealand Animal Ethics Committee (permit no. 181). This study used samples from six sites, representing five of the 13 ESUs described by Fouquet *et al.* [10]: the Brynderwyn Range; Northern, Central and Southern Coromandel; and Otawa (Figure 1). Genomic DNA was extracted using an AquaPure Genomic Tissue Kit (Bio-Rad), following the manufacturer’s protocol, and stored at −20°C.

### Microsatellite markers

A total of 168 individuals (Figure 1) were genotyped for 11 polymorphic microsatellite loci following the reaction and thermocycling protocols of Clay et al. [36]. Full microsatellite genotyping protocols, including multiplex details, are provided in Additional file 1: Supplementary Methods section “Microsatellite genotyping”.

### Characterization and genotyping of MHC class II DAB gene

To characterize *L. hochstetteri* MHC sequences, we generated a spleen transcriptome, sequenced on a Roche GS Junior 454 Sequencer (Landcare Research, Auckland). These data allowed us to sequence a 745-bp fragment of MHC IIB (designated DAB; Genbank accession: KP892996) incorporating partial exon 2 through to the 3’ UTR, as predicted from alignment with *X. laevis* MHC class II beta sequence (Genbank accession: D13684 [37]). Full details of the specific protocol used to identify and characterize *L. hochstetteri* MHC DAB are provided in Additional file 1: Supplementary Methods section “Transcriptome sequencing and MHC class II DAB characterization′.

To assay population levels of diversity, PCR primers (LehoIIBUpper: 5΄− GCGAAGTCTCAGTGTT −3΄ and LehoIIBLower: 5΄− CTTGTCTACAGTGTAAGGTT −3΄) were designed using Oligo6 (Molecular Biology Insights, Inc), targeting a 249–252 bp fragment within exon 2 of the MHC DAB gene (full length of exon 2 predicted to be 282 bp). These primers were designed to anneal to the most evolutionarily conserved regions, as predicted from multiple sequence alignments with anuran class II beta genes, while including the maximal number of putative peptide binding sites predicted from *X. laevis* MHC class II beta sequence. We carried out two PCRs per individual and cloned these using the pGEM-T Easy Vector System II (Promega), following the manufacturer’s recommendations. PCRs were carried out at Landcare Research Auckland laboratories (New Zealand) prior to export of the synthetic DNA products to the Australian Wildlife Genomics Group laboratories at the University of Sydney (Australia) where all further protocols were carried out. Full PCR and cloning methods are provided in Additional file 1: Supplementary Methods section “Amplification and cloning”). Twelve clones per PCR product were sequenced using the T7 primer at the Australian Genome Research Facility, Ltd (AGRF, Sydney, Australia). A maximum of two allele variants was obtained per individual, indicating that our primers were specific for a single locus. A clone sequence was accepted as a DAB allele if it was isolated from two independent PCR reactions (either two reactions from the same individual, or from two different individuals).

### Polymorphism analyses

Cloned DAB sequences were checked for quality, trimmed, and aligned using ClustalW, all within BioEdit v7 [38]. For each individual, duplicate sequences were summarized into genotypes with two alleles retained for analysis. Individuals that either returned new alleles (previously unobserved in other individuals) or more than two alleles from the first PCR product were retyped using the second PCR product. All except one individual were confirmed to have no more than two alleles using this cloning and sequencing approach. One individual from Northern Coromandel (sample ID: CorC327) showed 4 alleles as isolated from 2 independent PCR products. As this could either represent contamination of the DNA sample or duplication of the DAB gene within the individual, we removed the individual from further analysis.

DAB sequence polymorphism statistics (haplotype diversity, *Hd*; number of polymorphic sites, *S*; nucleotide diversity, π; average number of nucleotide differences, *k*) were calculated using DnaSP v4.10 [39]. GenAlEx v6.5 [40] was used to calculate microsatellite polymorphism statistics (observed, *H*_*O*_, and expected heterozygosity, *H*_*E*_). Arlequin v3.5 [41] was used to test for Hardy Weinberg Equilibrium for both microsatellite marker and DAB genotype data, and used to test for linkage disequilibrium between microsatellite markers. For both marker types, allelic richness for each ESU was calculated using FSTAT v2.9.3.2 [42]. To control for differences in sampling sizes, 95% CIs of the expected number of alleles based on sample size alone were calculated using permutation tests in R 3.0.2 (R Core Team). To investigate the contribution of genetic drift on the DAB gene, we performed a linear regression for allelic richness of microsatellite markers and the DAB gene in R, under the assumption that our microsatellite loci were selectively neutral.

Traditional measures of genetic differentiation such as *F*_*ST*_ and *G*_*ST*_ can give misleading results when calculated for highly polymorphic genes, such as the MHC [43]. These statistics approach zero when gene diversity is high even when populations are completely differentiated (no shared alleles). As such, using *F*_*ST*_ to compare populations for microsatellites, loci which have limited variability, versus our MHC gene, which has extremely high variability, would be inappropriate. Therefore, we used *G’*_*ST*_ [44] and *D*_*EST*_ [43] to measure both MHC and microsatellite differentiation between our populations, calculated using SMOGD 1.2.5 [45]. Isolation by distance at each marker type was investigated by Mantel tests as implemented by the ade4 1.6-2 package in R.

### Tests of recombination and positive selection

A global test for positive selection on MHC was carried out in MEGA using a codon-based Z test for selection across the DAB alleles (test statistic: *d*_*N*_-*d*_*S*_). We used the HyPhy package [46] implemented on the Datamonkey webserver [47] for model selection, to test for recombination and to detect sites under selection. The model selection tool [48] was used to identify the optimal nucleotide substitution model for further analyses. Evidence for recombination among *L. hochstetteri* DAB alleles was detected using single breakpoint (SBP) analysis using small sample AIC (AIC_C_) [49]. Recombination was taken into account in the implementation of three separate models of codon-based positive selection: single-likelihood ancestor counting (SLAC) [50], random-effects likelihood approach (REL) [50], and mixed effect model of evolution (MEME) [51], which use different methods to detect sites under selection. We adopted a conservative approach whereby amino acid sites identified by two or more models were retained as sites under positive selection for further analyses.

### Identification of DAB supertypes

Amino acid sites under positive selection as identified above were used for cluster analysis to define DAB supertypes, following Doytchinova and Flower [52]. All other amino acid sites (i.e. those that were not found to evolve under positive selection) were excluded during supertype definition. Each retained site was characterized according to five physiochemical descriptor variables: z1 (hydrophobicity), z2 (steric bulk), z3 (polarity), z4 and z5 (electronic effects) [53]. Discriminant analysis of principle components (DAPC) was implemented to define DAB gene clusters using adegenet 1.4-0 package in R [54,55]. This analysis implements a *k*-means clustering algorithm based on Bayesian Information Criterion (BIC); we used a ∆BIC ≤2 to identify optimal numbers of clusters. DAPC was then performed on retained principal components to assign *LehoDAB* alleles to a supertype. Population differentiation based on supertypes was estimated using SMOGD and isolation by distance analyzed by Mantel testing, as described above.

## Results

### Genetic diversity at microsatellite markers and MHC class II-DAB

Two microsatellite markers (Lhoc10 and Lhoc26) showed variable amplification success across sampled ESUs and were therefore removed from the analysis. All individuals were successfully genotyped at the remaining nine microsatellite markers. A total of 34 alleles were observed (2–5 alleles per marker). The microsatellite markers showed an average observed heterozygosity of 0.164 across the ESUs (ranging from 0.073-0.327). We observed eight occurrences where microsatellite allele frequencies deviated from Hardy-Weinberg Equilibrium (HWE) but these were not statistically significant after Holm-Bonferroni correction for multiple comparisons (Additional file 2: Table S1). From 36 comparisons, linkage disequilibrium was observed between six microsatellite marker pairs, but these were not statistically significant after Holm-Bonferroni correction.

At the MHC class II-DAB locus, 74 sequence variants (hereafter referred to as “alleles”) were characterized from 121 *L. hochstetteri* samples (Genbank accessions: KP892997-KP893070). DAB alleles were between 216–219 nucleotides, encoding a 72–73 amino acid product that varied in length on account of a single amino acid indel at position 69. These 74 alleles contained 76 polymorphic nucleotide sites with an average of 19.6 nucleotide differences (*k*), gene diversity (*Hd*) of 0.958, and nucleotide diversity (π) of 0.0906 (Table 1). Global tests for positive selection provided evidence for historical selection on the DAB region with a significant test statistic (*d*_*N*_*-d*_*S*_) of 2.08 (*P-*value = 0.020). Lower DAB diversity was observed in the Southern Coromandel and Otawa ESUs and lower microsatellite diversity was observed in Northern Coromandel, Southern Coromandel and Otawa (Table 1). By linear regression analysis, we observed a correlation between allelic richness of microsatellites and DAB (slope = 12.085; *P-*value = 0.009, *N* = 5 populations).

**Table 1:** **Summary polymorphism statistics for MHC class II-DAB and microsatellite markers**

	**DAB beta 1 domain**										**Microsatellites**						
**Population**	***N***	***A***	**95% CI** ***N***_***A***_	***A***_***R***_	***H***_***O***_	***H***_***E***_	***Hd***	***S***	**π**	***k***	***N***	***Polymorphic Markers (/9)***	***A***	**95% CI** ***N***_***A***_	***A***_***R***_	***H***_***O***_	***H***_***E***_
Brynderwyn Range	18	14	(13–23)	14	0.389	0.900	0.925	57	0.0928	20.04	19	9	2.33 (2–4)	(2.11-3.00)	2.33	0.327	0.361
Northern Coromandel	41	29	(28–42)	18.7	0.268	0.918	0.929	63	0.0878	18.96	59	9	2.78 (2–5)	(2.89-3.56)	2.28	0.141	0.195
Central Coromandel	22	21	(16–27)	19.6	0.364	0.941	0.963	55	0.0861	18.6	29	7	2.67 (1–5)	(2.44-3.33)	2.49	0.203	0.221
Southern Coromandel	21	11	(16–26)	10.4	0.238	0.773	0.767	56	0.0804	17.36	35	6	1.89 (1–3)	(2.56-3.33)	1.72	0.073	0.116
Otawa	19	2	(14–24)	2	0.105	0.100	0.102	23	0.0108	2.36	26	1	1.11 (1–2)	(2.33-3.22)	1.11	0.073	0.054
Total Data Estimates	121	74		23.9	0.273	0.726	0.958	76	0.0906	19.58	168	6.4	3.78		2.63	0.164	0.189

Number of individuals, *N*; average number of alleles per locus, *A*; 95% confidence interval for expected number of alleles for given sample size, 95% CI *N*_*A*_; allelic richness, *A*_*R*_; observed heterozygosity, *H*_*O*_, and expected heterozygosity, *H*_*E*_; haplotype (gene) diversity, *Hd*; number of polymorphic (segregating) sites, *S*; nucleotide diversity, π; average number of nucleotide differences, *k,* and polymorphic markers describing the number of microsatellite markers that displayed polymorphism per population.

Statistically significant homozygous excess (*P*-values <0.001) was observed at the DAB locus for all ESUs, except Otawa (*P*-value = 1). We could not rule out the presence of null alleles in these four ESUs. If null alleles are present, they may indicate a single common allele or cluster of alleles that were not amplified by our DAB primers, despite employing a low annealing temperature during PCR (53°C) and designing primers for conserved regions of the DAB exon 2. If the homozygote excess is due to null alleles, there may be more DAB alleles present in Brynderwyn, Northern Coromandel, Central Coromandel, and Southern Coromandel, in excess to those characterized here. The Otawa population appeared to be in Hardy-Weinberg equilibrium.

### Recombination and Positive selection

**Figure 2:**
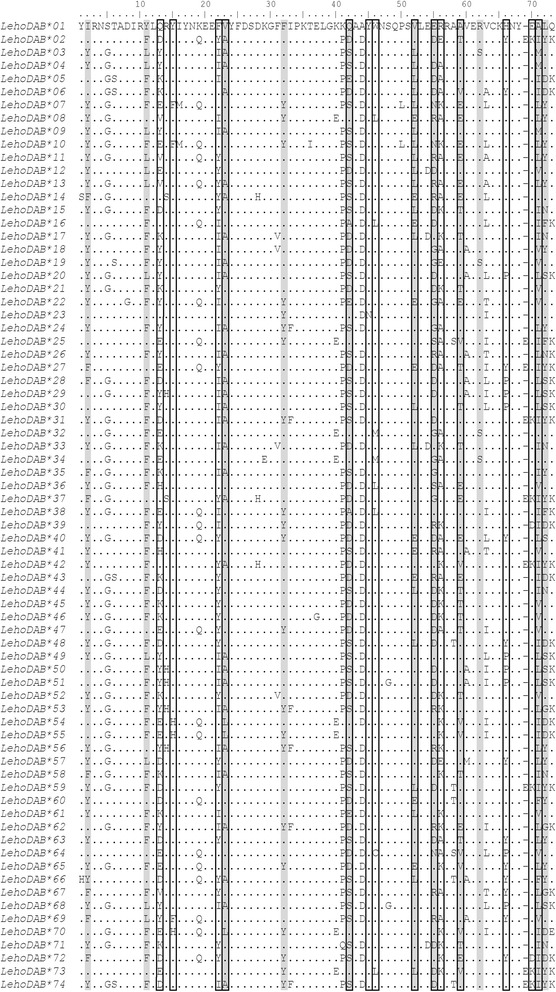
Amino acid alignment of MHC class II-DAB alleles characterized in *Leiopelma hochstetteri*. Positively selected sites identified by at least two models (SLAC/REL/MEME; see Methods), shaded in grey; putative peptide binding sites predicted in *Xenopus laevis* [37] boxed in black outline.

Recombination was detected in the DAB sequence dataset with strong AIC_C_ support for breakpoint located at nucleotide site 162. With this recombination taken into account, positive selection was detected at 11 sites from at least two tests (SLAC/REL/MEME) (Table 2). Six of these sites overlapped with peptide binding residues predicted from alignment with *Xenopus laevis* [37] (Figure 2).

**Table 2:** **Summary of significant results from likelihood models for amino acid sites under selection**

	**SLAC**		**REL**		**MEME**	
Amino acid site	Normalized dN-dS	*P*-value	E[dN-dS]	Bayes Factor (dN > dS)	beta2	*P*-value
2	9.02	0.016	7.44	5111.36	14.88	0.004
**11**			7.46	1590.05	4.68	0.015
13					25.51	0.021
22					6.46	0.016
**23**	4.01	0.026	7.22	147.83	5.28	0.005
**32**			7.33	134.56	4.29	0.023
41					33.73	0.011
**42**	7.09	0.043	7.43	1743.83	73.74	<0.001
**52**	7.18	0.002	7.49	1.57E + 06	9.54	<0.001
**55**	6.34	0.042	7.48	3.02E + 06		
56					73.31	<0.001
**59**	7.61	<0.001	7.53	1.68E + 06	8.85	<0.001
60					3.68	0.031
**63**	7.62	<0.001	7.53	6.83E + 05	9.40	<0.001
**71**	4.79	0.010			22.20	0.026
**72**			7.19	205.14	33.54	<0.001
73					4.71	0.028

Amino acids with *P*-values <0.05 for SLAC/REL/MEME and Bayes Factor >100 for REL were considered to be under positive selection. Amino acid sites identified under selection from at least two models tested in bold.

### Differentiation between populations

Genetic differentiation estimated across microsatellite markers ranged from *G’*_*ST*_ 0.077-0.587 and *D*_*EST*_ 0.039-0.397 (Table 3). Otawa showed the highest levels of differentiation from all other ESUs (*D*_*EST*_ 0.174-0.397), while the four ESUs from the Coromandel Peninsula displayed the lowest differentiation from one-another (*D*_*EST*_ 0.039-0.098). For MHC, ESUs were highly differentiated at the DAB locus, with only three alleles (4.1%) observed in more than one ESU (LehoDAB*49 and LehoDAB*55 present in Northern Coromandel and Central Coromandel; LehoDAB*73 present in Southern Coromandel and Otawa). Correspondingly, population differentiation measures for the DAB locus were very high (Table 3). Northern Coromandel and Central Coromandel also shared amino acid products encoded by different sequence variants (LehoDAB*15 and LehoDAB*44; LehoDAB*21 and LehoDAB*45; and LehoDAB*29 and LehoDAB*50).

**Table 3:** **Population differentiation across 5 ESUs at microsatellite markers, MHC class II DAB gene and MHC class II-DAB supertypes**

**A: Microsatellites**	**Brynderwyn Range**	**Northern Coromandel**	**Central Coromandel**	**Southern Coromandel**	**Otawa**
Brynderwyn Range		0.115	0.188	0.243	0.174
Northern Coromandel	0.168		0.049	0.098	0.235
Central Coromandel	0.254	0.086		0.039	0.336
Southern Coromandel	0.319	0.142	0.077		0.397
Otawa	0.267	0.296	0.401	0.587	
**B: MHC II-DAB**					
Brynderwyn Range		1	1	1	1
Northern Coromandel	1		1	1	1
Central Coromandel	1	1		0.735	1
Southern Coromandel	1	1	0.748		0.995
Otawa	1	1	1	0.997	
**C: MHC II-DAB supertypes**					
Brynderwyn Range		0.450	0.365	0.162	0.835
Northern Coromandel	0.101		0.044	0.459	0.248
Central Coromandel	0.091	0.012		0.357	0.200
Southern Coromandel	0.043	0.116	0.101		0.567
Otawa	0.397	0.166	0.151	0.337	

*D*_*EST*_ above diagonal (Jost 2008); *G’*_*ST*_ below diagonal (Hedrick 2005), microsatellite data are averaged across 9 loci.

**Figure 3:**
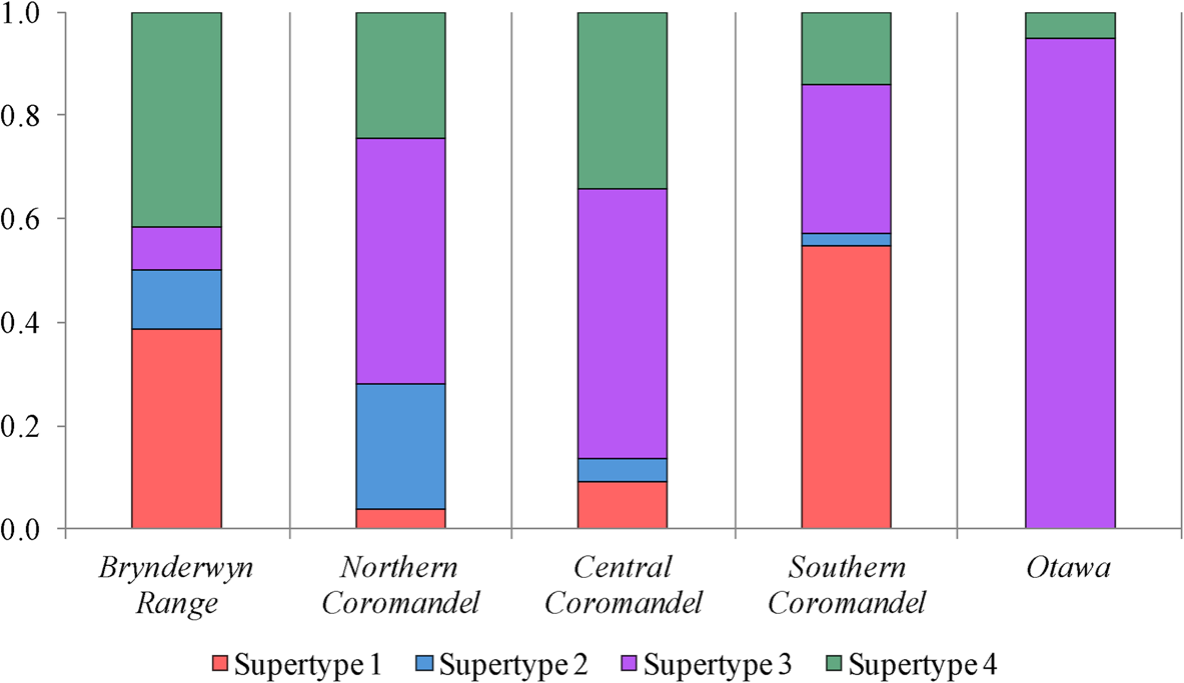
Supertype frequencies in five *Leiopelma hochstetteri* ESUs.

DAPC analysis revealed an optimum of four supertype clusters based on a ΔBIC ≤ 2 (Additional file 3: Figure S1). Each supertype cluster contained between 10–33 DAB alleles (Additional file 2: Table S2). All supertypes were represented in every ESU, except Otawa, which had only two *LehoDAB* alleles present (Figure 3). Interestingly, these two alleles were assigned to different supertypes, implying that this ESU, despite having relatively low MHC diversity, does have some functional variability present. Population differentiation at the level of DAB supertype ranged from 0.044-0.835 (*D*_*EST*_) and 0.012-0.397 (*G’*_*ST*_) (Table 3). The Northern and Central Coromandel ESUs showed the lowest supertype differentiation from one-another, with *D*_*EST*_ of 0.044 and *G’*_*ST*_ of 0.012. Mantel tests found no significant association between genetic differentiation and geographic distance at microsatellite markers, MHC or MHC supertypes (Additional file 2: Table S3).

Although we implemented a conservative approach to identifying 11 positively selective sites for our supertyping analysis, we note that the MEME approach of predicting sites under pervasive versus episodic selection might be more appropriate to infer balancing selection on the MHC. As such, we repeated our supertyping analysis using the 16 sites predicted under just MEME, for comparison. Similar to our main analysis, the MEME-only results gave three supertypes, which were similarly represented in each population with the exception of Otawa, which contained two supertypes (Additional file 3: Figure S2). Population differentiation estimates were qualitatively similar to our main analysis, with lower differentiation between ESUs on the Coromandel Peninsula (Additional file 2: Table S4).

## Discussion

We characterized a MHC II-DAB locus in this threatened species, with 74 alleles identified across five populations. MHC diversity present in wild populations is the result of the interplay between neutral evolutionary forces, such as genetic drift and gene flow, and selective forces such as mate choice and host-pathogen co-evolution [16,17,56,57]. Generally, positive selection may act on beneficial mutations arising within the MHC, and this variation is hypothesized to be maintained by selective forces mediated by pathogen variability and disassortative mating [16,56,57]. We found evidence for positive selection acting on the DAB in *L. hochstetteri* and extreme population differentiation between ESUs: every population was nearly unique in their DAB sequence variation. In four of our five populations, we found a high excess of homozygosity; we cannot rule out null alleles as a possible driver of this pattern. Nevertheless, the patterns we observed at the detected alleles are still informative for inferring patterns of diversity and selection across populations.

As selection acts on phenotypes, not genotypes, we examined the functional properties of the DAB alleles characterized in *L. hochstetteri*, to investigate whether ESUs retained similar DAB peptide binding functions despite lacking shared DAB alleles. Supertyping approaches have been employed in several other MHC studies in wild populations for both studying population diversity and for investigating associations between MHC supertypes and disease [34,35,58,59]. We identified four DAB supertypes that were all represented in each ESU, excluding Otawa, which had only two supertypes present. Despite nearly all sites sharing the same DAB supertypes, considerable population differentiation was observed, resulting from differences in supertype frequencies (Table 3; Figure 3). A notable exception to this pattern is the ESU pair of Northern and Central Coromandel, which were very weakly differentiated (Table 3).

In general, amphibians are regarded to be poor dispersers with high site fidelity [60,61]. *Leiopelma hochstetteri* is a habitat specialist; preferring rock piles in unsilted streambeds in which they have micro-territories [4] with limited daily movements [12]. Dispersal behavior and ability within the species is still unknown; however the extreme genetic structuring seen at adaptive genetic markers (current study), neutral genetic markers [7,10] and cytogenetic distinctions [9,62] does imply minimal dispersal. If this study were expanded to other *L. hochstetteri* ESUs, it is likely that additional DAB alleles would be characterized, and that each ESU could have a unique pattern of DAB variation with very few shared DAB alleles. If ESUs lack connectivity, each unit would experience distinct pathogen diversities over their evolutionary histories and the spatially and/or temporally variable selection would contribute to the observed differentiation of DAB supertypes [18]. The low differentiation observed between Northern and Central Coromandel for DAB supertype suggests the presence of similar environmental factors across the continuous habitat of the Coromandel Peninsula, resulting in similar selective pressures in both ESUs. We are unaware of any studies of disease prevalence in *L. hochstetteri* on the Coromandel Peninsula, but such investigation would shed more light into the potential pathogen-mediated selection pressures.

We observed a correlation between allelic richness of microsatellites and DAB, implying the contribution of genetic drift in shaping MHC variation in our studied ESUs (Table 1). Notably, the Southern Coromandel and Otawa ESUs possess lower genetic diversity at both marker types, compared to the other ESUs. This may reflect the predominance of genetic drift over balancing selection acting on the MHC in these ESUs. In particular, Otawa had the lowest DAB diversity of all studied ESUs: only two allele variants were present, with the predominant allele (LehoDAB*74) occurring at a frequency of 0.947, and the alternate allele (LehoDAB*73) only present in two heterozygotes (n = 19 individuals). This could reflect strong directional selection acting in this ESU drawing the LehoDAB*74 allele close to fixation, or a depletion in DAB diversity due to population decline or bottleneck. A meta-analysis of the relative roles of genetic drift and balancing selection on MHC variation revealed that there is may be greater loss of MHC than neutral diversity during population bottlenecks [21]. Furthermore, simulations have shown that balancing selection acting in small populations can deplete MHC variation faster than drift [63].

We suggest that the Otawa ESU, with its limited MHC variation, may be at a greater risk from disease outbreaks. Investigations into *L. hochstetteri* disease and pathogens are lacking, but have attracted high priority in the recently published native frog recovery plan [1] and would greatly improve our understanding of the contribution of MHC variation to pathogen resistance/susceptibility in this species. The DAB gene is not, however, the only locus involved in pathogen recognition and immune response [64], and further immunogenetic investigation into other functionally significant genes, such as toll-like receptors or anti-microbial peptides, in this population will improve our risk estimates. A recent assessment of the threat status of *Leiopelma* spp proposed that the Otawa *L. hochstetteri* population was conservation dependent [5]. Human interventions, such as translocation of individuals with DAB alleles spanning different supertypes to this site, may increase genetic diversity. However, our results do not rule out the possibility that limited DAB diversity in this ESU represents local adaptation; close genetic monitoring of translocation success could reveal this.

*Leiopelma hochstetteri* provides an important benchmark for future MHC studies in other *Leiopelma* species, which are substantially more vulnerable, with fewer numbers and fewer populations [1]. *Leiopelma archeyi* occurs in two natural populations on the New Zealand North Island: on the Coromandel Peninsula and in the Whareorino forest [65,66]. Between 1996 and 2001 the Coromandel population experienced a rapid decline [67] and is now persisting at severely reduced numbers. *Leiopelma pakeka* occurs in a stable, natural population in remnant forest on Maud Island, and in two introduced populations, in another habitat on Maud Island and on Motuara Island [68,69]. *Leiopelma hamiltoni* is found in a single natural population in a small rock tumble on Stephens Island, with a population estimate of only 300 mature individuals, and an introduced population established in 2004 on Nukuwaiata Island [1,70-72]. The MHC primers developed herein could be used in these related species to help understand functional genetic variation in these populations and assist in translocation planning and monitoring to ensure adequate supertype variation is retained in both donor and translocated populations. Longitudinal studies could provide insights into selective pressures acting in introduced populations, where frog-naïve environments may not harbor co-evolved pathogens. Finally, cross-species studies would be valuable for identifying long-term selection pressures that may have shaped MHC diversity within the ancient *Leiopelma* genus.

## Conclusions

Our study found high MHC-DAB allelic diversity in *L. hochstetteri* as a result of positive selection and extremely high population differentiation. Nearly every population possessed a unique DAB allele pool. DAB- supertype differentiation was high among ESUs suggesting that selection pressures vary spatially and/or temporally. Northern and Central Coromandel were exceptions to this, with lower differentiation of DAB supertype frequencies, which may imply similar selective pressures as a result of shared environmental characteristics. Very low DAB diversity in Otawa, with only two alleles present, may contribute to a greater extinction risk from disease outbreaks in this ESU.

## Availability of supporting data

Nucleotide sequences for partial MHC class II beta transcript and the 74 DAB alleles are available on Genbank (Accessions numbers: KP892996 - KP893070).

## Additional files

Additional file 1:
**Supplementary methods, including table of microsatellite marker multiplex details.**
Additional file 2:
**Supplementary Tables S1-S4.**
Additional file 3:
**Supplementary Figures S1-S2.**

## Abbreviations

MHC: Major histocompatibility complex
ESU: Evolutionarily Significant Unit
IUCN: International Union for Conservation of Nature
DAB: MHC class II beta gene
PCR: Polymerase chain reaction
SLAC: Single-likelihood ancestor counting
REL: Random-effects likelihood approach
MEME: Mixed effect model of evolution
SBP: Single breakpoint
EDGE: Evolutionarily distinct and globally endangered
DAPC: Discriminant analysis of principle components
BIC: Bayesian information criterion
