# Supplementary material for: Selection on MHC class II supertypes in the New Zealand endemic Hochstetter’s frog
Source: BMC Evol Biol. 2015 Apr 13;15:63. doi: 10.1186/s12862-015-0342-0 (PMC4415247; doi:10.1186/s12862-015-0342-0)
Supplement: Additional file 1: — Supplementary methods, including table of microsatellite marker multiplex details. [file 12862_2015_342_MOESM1_ESM.pdf]

Supplementary Methods

Microsatellite genotyping

Individuals were genotyped for 11 microsatellites markers using two multiplexes (Supplementary Methods Table 1). Polymerase chain reaction (PCR) amplifications were performed in 10 µl reactions containing 1× PCR buffer with $MgCl_2$ (50 mM Tris/HCl, 10 mM KCl, 5 mM $[NH_4]_2SO_4$, 2 mM $MgCl_2$, pH 8.3), 0.4 µl BSA (10 mg/ml), 200 µM of each dNTP, 0.1-0.4 µM of each primer, 0.8U of FastStart Taq DNA Polymerase (Roche Diagnostics), and approximately 20 ng of DNA template. Amplification conditions on a GeneAmp PCR System 9700 thermocycler (Applied Biosystems) were: initial denaturation at 95°C for 4 min; 38 cycles of 30 s at 94°C, 30 s at 60°C, 45 s at 72°C; and a final extension of 40 min at 72°C. PCR products were separated using capillary electrophoresis with GeneScan 500 Liz size standard (Applied Biosystems) on an Applied Biosystems 3130xl Genetic Analyzer and scored using GeneMapper v4.0 (Applied Biosystems).

Transcriptome sequencing and MHC class II DAB characterization

A *L. hochstetteri* spleen transcriptome was sequenced on a Roche GS Junior 454 Sequencer (Landcare Research, Auckland). We obtained 46,369 reads with an average length of 322 base pairs (bp). Geneious 5.4 (Biomatters) was used to assemble reads into 4,811 contigs, which in turn were used to generate a custom BLAST database (NCBI). *Xenopus laevis* MHC class II (Genbank accession: DQ268506) was used to query the custom blast database, identifying contigs with significant e-values (less than 0.05). Contigs were used as queries in tblastx searches against the full NCBI nucleotide collection (nr/nt) and only one (Contig_1086) was significantly similar to MHC class II beta of other anuran species (*X. laevis*, *Silurana tropicalis*, and *Quasipaa spinosa*) and a caudate species (*Andrias davidianus*). Alignment with *X. laevis* MHC class II beta gene sequence (Genbank accession:

D13684) predicted that this contig represented the complete coding sequence (Sato *et al.* 1993).

PCR primers were designed using Oligo 6 (Molecular Biology Insights, Inc) to amplify a 920 bp fragment of the open reading frame (primer pair: LehoMHCIIB-F1 5′-GCACTTCCCTCTGTCAGACATC3′ and LehoMHCIIB-R1 5′-CCTGGGCTTGGTCATAATTAAA -3′) to confirm the sequence of this contig. PCR reactions were carried out in 25 µl volumes, containing 1× FastStart High Fidelity Reaction Buffer with $MgCl_2$ (Roche), 200 µM dNTPs, 400 nM each primer, 1 U FastStart High Fidelity Enzyme Blend (Roche) and approximately 20 ng of DNA template. Amplification was achieved using thermal cycling conditions of initial denaturation and activation of the polymerase at 95°C for 4 minutes; 38 cycles of 30 s at 94°C, 30 s at 53°C, 60 s at 72°C; and a final extension at 72°C for 7 minutes. Direct sequencing of purified products was carried out with BigDye™ Terminator v3.1 (Applied Biosystems) following the manufacturer's protocol. Sequences were analyzed on an Applied Biosystems 3130xl Genetic Analyzer using DNA Sequencing Analysis Software v5.3.1 (Applied Biosystems). Resulting DNA sequences were compared and edited manually using the program Sequencher v4.6 (Gene Codes). The final trimmed sequence (745 bp; Genbank Accession: KP892996) included partial exon 2 through to the 3’ UTR and shared 95.4% sequence identity to Contig_1086, confirming the predicted MHC sequence from the transcriptome assembly.

Amplification and cloning

Two PCR reactions per individual were carried out as for MHC class II DAB characterization (above), except with an annealing temperature of 60°C. Amplification of the target fragment was confirmed by running PCR products alongside molecular weight ladder HyperLadder IV (Bioline) on a 1.6% agarose 1× TBE gel, stained with 1× *SYBR Safe* DNA gel stain (Invitrogen), at 120 V for approximately 20 minutes. PCR products were excised from the

gels and purified using QIAEX II Gel Purification Kit (QIAGEN) according to the manufacturer's specifications. Purified PCR products were ligated into a pGEM-T Easy plasmid vector (Promega) using an 8:1 product: vector ratio and overnight incubation at 4°C; 5 µl of ligation product was used in the transformation of JM109 Competent *Escherichia coli* cells (Promega). Cells were plated onto LB Agar plates with 100 µg/ml *ampicillin (Sigma)*, 0.5 mM IPTG (Astral Scientific) and 80 µg/ml Xgal (Promega) to facilitate blue/white screening, and incubated overnight 37°C. Twelve clones per PCR product and per individual were cultured and grown overnight in LB broth with 50 µg/ml *ampicillin (Sigma)*. Plasmids were purified using either QIAprep Spin Miniprep Kit (QIAGEN) or DirectPrep 96 Miniprep Kit (QIAGEN) following the manufacturer's specifications. Direct sequencing of purified plasmids was performed using T7 primer.

Supplementary Methods Table 1. Multiplex design for microsatellite marker polymerase chain reaction. Microsatellite loci described in Clay *et al.* (2010).

Multiplex	Locus	5' Fluorescent label	Fragment sizes (bp)
1	*Lhoc*07	VIC	131 - 143
	*Lhoc*11	NED	158 - 170
	*Lhoc*15	PET	173 - 183
	*Lhoc*23	NED	144 - 152
	*Lhoc*25	VIC	190 - 202
	*Lhoc*26	PET	210 - 228
2	*Lhoc*05	NED	118 - 130
	*Lhoc*08	PET	151 - 157
	*Lhoc*10	FAM	153 - 161
	*Lhoc*13	VIC	168 - 178
	*Lhoc*19	NED	175 - 205
