# Supplementary material for: Selection on MHC class II supertypes in the New Zealand endemic Hochstetter’s frog
Source: BMC Evol Biol. 2015 Apr 13;15:63. doi: 10.1186/s12862-015-0342-0 (PMC4415247; doi:10.1186/s12862-015-0342-0)
Supplement: Additional file 2: — Supplementary Tables S1-S4. [file 12862_2015_342_MOESM2_ESM.pdf]

Supplementary Tables

Supplementary Table 1. Summary of Hardy-Weinberg results for 9 polymorphic microsatellite markers for each ESU. Observed heterozygosity, H_O, and expected heterozygosity, H_E. Microsatellite markers deviating from Hardy-Weinberg Equilibrium expectations indicated in bold; * indicates non-significance after Bonferroni correction for multiple tests

ESU	Locus	H_O	H_E	*P*-value	s.d.
Brynderwyn Range	***Lhoc*05**	**0.526**	**0.626**	**0.0191***	**0.0001**
	*Lhoc*07	0.368	0.462	0.6075	0.0005
	*Lhoc*08	0.053	0.053	1	0
	*Lhoc*11	0.263	0.235	1	0
	*Lhoc*13	0.211	0.193	1	0
	*Lhoc*15	0.158	0.235	0.2588	0.0004
	*Lhoc*19	0.737	0.738	0.1099	0.0003
	*Lhoc*23	0.263	0.422	0.1246	0.0003
	*Lhoc*25	0.368	0.371	1	0
Northern Coromandel	*Lhoc*05	0.305	0.375	0.0622	0.0003
	***Lhoc*07**	**0.203**	**0.347**	**0.0033***	**0.0001**
	*Lhoc*08	0.119	0.113	1	0
	*Lhoc*11	0.034	0.034	1	0
	*Lhoc*13	0.136	0.160	0.3390	0.0005
	***Lhoc*15**	**0.102**	**0.159**	**0.0284***	**0.0002**
	***Lhoc*19**	**0.153**	**0.249**	**0.0113***	**0.0001**
	*Lhoc*23	0.017	0.017	1	0
	***Lhoc*25**	**0.203**	**0.321**	**0.0071***	**0.0001**
Central Coromandel	*Lhoc*05	0.310	0.376	0.2406	0.0004
	*Lhoc*07		Monomorphic		
	*Lhoc*08	0.103	0.160	0.1703	0.0004
	*Lhoc*11	0.345	0.336	0.1193	0.0003
	*Lhoc*13	0.172	0.160	1	0
	*Lhoc*15		Monomorphic		
	*Lhoc*19	0.172	0.195	0.2516	0.0005
	*Lhoc*23	0.172	0.163	1	0
	*Lhoc*25	0.552	0.635	0.4946	0.0005
Southern Coromandel	*Lhoc*05	0.429	0.581	0.0915	0.0003
	*Lhoc*07	0.029	0.029	1	0
	*Lhoc*08		Monomorphic		
	*Lhoc*11	0.057	0.056	1	0
	***Lhoc*13**	**0.000**	**0.056**	**0.0150***	**0.0001**
	*Lhoc*15		Monomorphic		
	***Lhoc*19**	**0.029**	**0.086**	**0.0150***	**0.0001**
	*Lhoc*23		Monomorphic		
	***Lhoc*25**	**0.114**	**0.248**	**0.0102***	**0.0001**
Otawa	*Lhoc*05		Monomorphic		
	*Lhoc*07		Monomorphic		
	*Lhoc*08		Monomorphic		
	*Lhoc*11		Monomorphic		
	*Lhoc*13		Monomorphic		
	*Lhoc*15		Monomorphic		
	*Lhoc*19	0.654	0.491	0.1140	0.0003
	*Lhoc*23		Monomorphic		
	*Lhoc*25		Monomorphic		

Supplementary Table 2. Supertype membership of *Leiopelma hochstetteri* MHC class II-DAB alleles

Supertype	*LehoDAB* allele
1	*LehoDAB*03*
	*LehoDAB*04*
	*LehoDAB*09*
	*LehoDAB*11*
	*LehoDAB*12*
	*LehoDAB*13*
	*LehoDAB*20*
	*LehoDAB*49*
	*LehoDAB*57*
	*LehoDAB*68*
	*LehoDAB*69*
2	*LehoDAB*08*
	*LehoDAB*14*
	*LehoDAB*16*
	*LehoDAB*22*
	*LehoDAB*27*
	*LehoDAB*40*
	*LehoDAB*41*
	*LehoDAB*43*
	*LehoDAB*60*
	*LehoDAB*65*
3	*LehoDAB*07*
	*LehoDAB*10*
	*LehoDAB*15*
	*LehoDAB*17*
	*LehoDAB*18*
	*LehoDAB*19*
	*LehoDAB*21*
	*LehoDAB*24*
	*LehoDAB*26*
	*LehoDAB*28*
	*LehoDAB*31*
	*LehoDAB*33*
	*LehoDAB*35*
	*LehoDAB*36*
	*LehoDAB*37*
	*LehoDAB*38*
3	*LehoDAB*42*
	*LehoDAB*44*
	*LehoDAB*45*
	*LehoDAB*46*
	*LehoDAB*48*
	*LehoDAB*52*
	*LehoDAB*53*
	*LehoDAB*56*
	*LehoDAB*58*
	*LehoDAB*59*
	*LehoDAB*61*
	*LehoDAB*63*
	*LehoDAB*66*
	*LehoDAB*67*
	*LehoDAB*71*
	*LehoDAB*72*
	*LehoDAB*74*
4	*LehoDAB*01*
	*LehoDAB*02*
	*LehoDAB*05*
	*LehoDAB*06*
	*LehoDAB*23*
	*LehoDAB*25*
	*LehoDAB*29*
	*LehoDAB*30*
	*LehoDAB*32*
	*LehoDAB*34*
	*LehoDAB*39*
	*LehoDAB*47*
	*LehoDAB*50*
	*LehoDAB*51*
	*LehoDAB*54*
	*LehoDAB*55*
	*LehoDAB*62*
	*LehoDAB*64*
	*LehoDAB*70*
	*LehoDAB*73*

Supplementary Table 3. Summary of Mantel test results of isolation by distance at microsatellite markers, the MHC class II-DAB gene and the DAB supertypes

Marker type	R^2	*P*-value
Microsatellites	0.224	0.329
DAB	0.356	0.199
DAB supertypes	0.445	0.107

Supplementary Table 4. Population differentiation across 5 ESUs at MHC class II-DAB supertypes generated from cluster analysis based on positively selected sites predicted only from Mixed Effect Model of Evolution (MEME)

	Brynderwyn Range	Northern Coromandel	Central Coromandel	Southern Coromandel	Otawa
Brynderwyn Range		0.175	0.026	0.327	0.808
Northern Coromandel	0.077		0.029	0.011	0.313
Central Coromandel	0.013	0.011		0.127	0.544
Southern Coromandel	0.129	0.004	0.045		0.190
Otawa	0.513	0.234	0.357	0.149	

D_{EST} above diagonal (Jost 2008); G'_{ST} below diagonal (Hedrick 2005)
