# Supplementary material for: Selection on MHC class II supertypes in the New Zealand endemic Hochstetter’s frog
Source: BMC Evol Biol. 2015 Apr 13;15:63. doi: 10.1186/s12862-015-0342-0 (PMC4415247; doi:10.1186/s12862-015-0342-0)
Supplement: Additional file 3: — Supplementary Figures S1-S2. [file 12862_2015_342_MOESM3_ESM.pdf]

Supplementary Figures

Supplementary Figure 1. Discriminant Analysis of Principal Components (DAPC) result to defining *Leiopelma hochstetteri* MHC class II-DAB supertypes

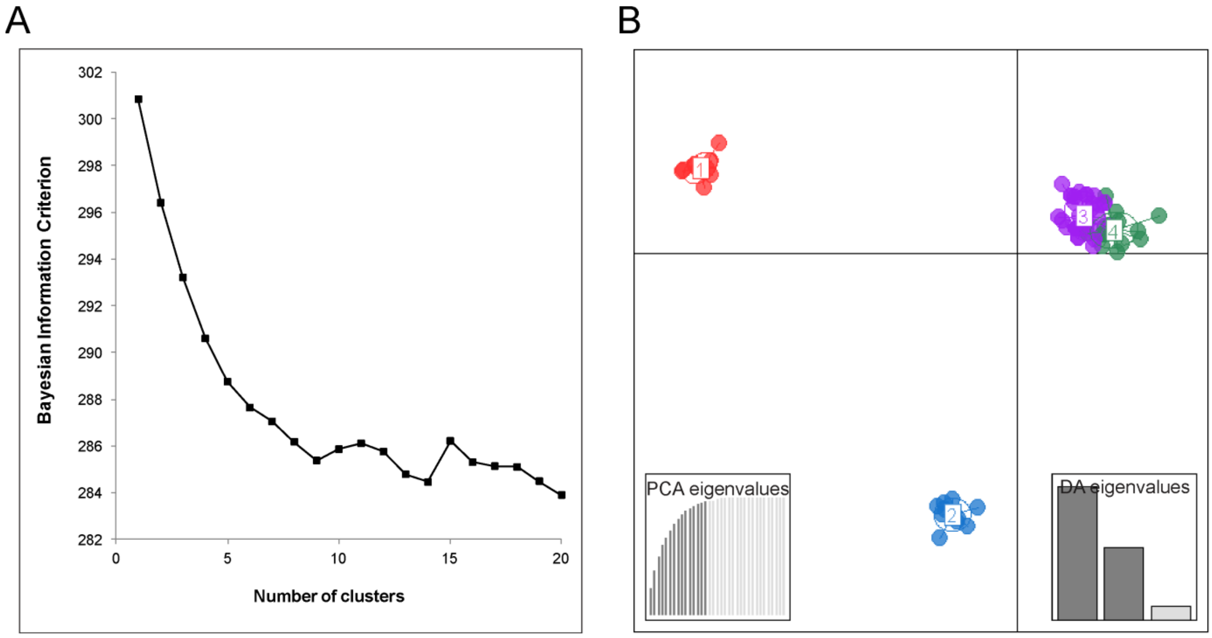

A. K-means clustering indicating number of *Leiopelma hochstetteri* MHC class II-DAB supertypes based on physiochemical descriptor variables for positive selected sites, as predicted by a consensus of at least two models of positive selection including single-likelihood ancestor counting (SLAC) (Pond & Frost 2005), random-effects likelihood approach (REL) (Pond & Frost 2005), and mixed effect model of evolution (MEME) (Murrell *et al.* 2012), with ΔBIC $\leq$2 to identify an optimal of 4 clusters. B. DAPC scatterplot of DAB supertypes; eigenvalues of the principle components analysis are displayed in lower left inset and eigenvalues of the discriminant analysis are displayed in lower right inset.

Supplementary Figure 2. Discriminant Analysis of Principal Components (DAPC) result to defining *Leiopelma hochstetteri* MHC class II-DAB supertypes using positively selected sites predicted by only Mixed Effects Model of Evolution

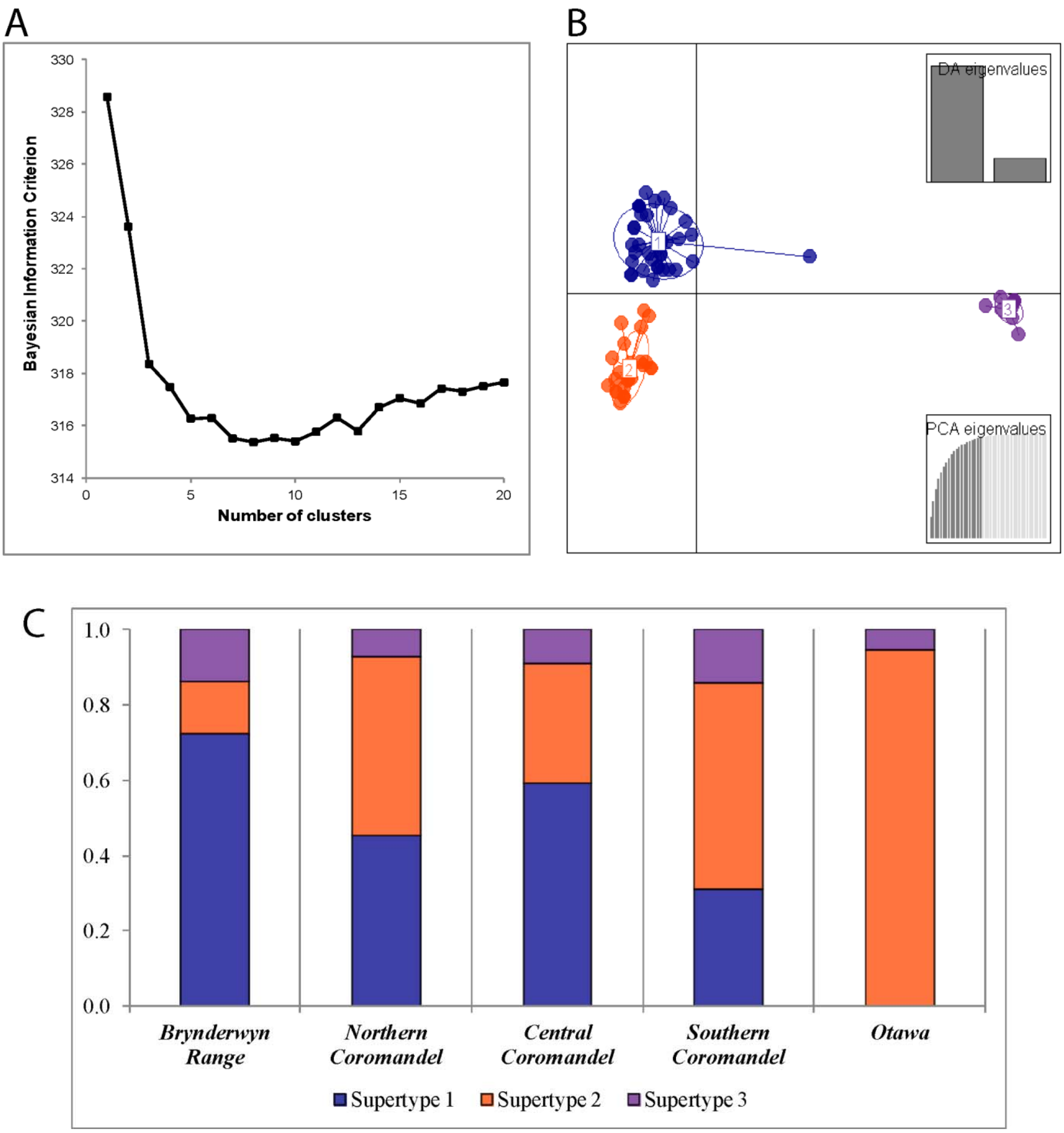

A. K-means clustering indicating number of *Leiopelma hochstetteri* MHC class II-DAB supertypes based on physiochemical descriptor variables for positive selected sites predicted by only mixed effect model of evolution (MEME) (Murrell *et al.* 2012), with ΔBIC ≤2 to identify an optimal of 3 clusters. B. DAPC scatterplot of DAB supertypes; eigenvalues of the principle components analysis are displayed in lower right inset and eigenvalues of the discriminant analysis are displayed in upper right inset. C. Supertype frequencies in five ESUs based on this clustering approach.
